# Relating the Surface Properties of Superparamagnetic Iron Oxide Nanoparticles (SPIONs) to Their Bactericidal Effect towards a Biofilm of *Streptococcus mutans*

**DOI:** 10.1371/journal.pone.0154445

**Published:** 2016-04-26

**Authors:** Taraneh Javanbakht, Sophie Laurent, Dimitri Stanicki, Kevin J. Wilkinson

**Affiliations:** 1 Department of Chemistry, University of Montreal, Montreal, Quebec, Canada; 2 Laboratory of NMR and Molecular Imaging, University of Mons, Mons, Belgium; 3 Center for Microscopy and Molecular Imaging (CMMI), Gosselies, Belgium; Brandeis University, UNITED STATES

## Abstract

This study was designed to determine the effects of superparamagnetic iron oxide nanoparticles (SPIONs) on the biological activity of a bacterial biofilm (*Streptococcus mutans*). Our hypothesis was that the diffusion of the SPIONs into biofilms would depend on their surface properties, which in turn would largely be determined by their surface functionality. Bare, positively charged and negatively charged SPIONs, with hydrodynamic diameters of 14.6 ± 1.4 nm, 20.4 ± 1.3 nm and 21.2 ± 1.6 nm were evaluated. Time-of-flight secondary ion mass spectrometry (TOF-SIMS) and electrophoretic mobility (EPM) measurements were used to confirm that carboxylic functional groups predominated on the negatively charged SPIONS, whereas amine functional groups predominated on the positively charged particles. Transmission electron microscopy (TEM) showed the morphology and sizes of SPIONs. Scanning electron microscopy (SEM) and EPM measurements indicated that the surfaces of the SPIONs were covered with biomolecules following their incubation with the biofilm. Bare SPIONs killed bacteria less than the positively charged SPIONs at the highest exposure concentrations, but the toxicity of the bare and positively charged SPIONs was the same for lower SPION concentrations. The positively charged SPIONs were more effective in killing bacteria than the negatively charged ones. Nonetheless, electrophoretic mobilities of all three SPIONs (negative, bare and positively charged) became more negative following incubation with the (negatively-charged) biofilm. Therefore, while the surface charge of SPIONS was important in determining their biological activity, the initial surface charge was not constant in the presence of the biofilm, leading eventually to SPIONS with fairly similar surface charges *in situ*. The study nonetheless suggests that the surface characteristics of the SPIONS is an important parameter controlling the efficiency of antimicrobial agents. The analysis of the CFU/mL values shows that the SPIONs have the same toxicity on bacteria in solution in comparison with that on the biofilm.

## Introduction

Nanoparticles (NPs) are small colloids (at least one dimension<100 nm) that have numerous environmental [1,2], medical and pharmaceutical applications [3,4]. They are also used frequently in sensors and for the diagnostics of chronic diseases [5]. Superparamagnetic iron oxide NPs (SPIONs) are a class of nanoparticles that have been widely used as drug delivery systems [6,7]. SPIONs have been reported to be effective for disrupting bacterial cell membranes [8][9]. Furthermore, their bactericidal effect appears to be increased when an AC magnetic field is applied, resulting in localized heating that is dependent upon the concentration of SPIONs [9]. For example, magnetically concentrated carboxyl-grafted SPIONs were shown to cause an approximately 8-fold higher bactericidal effect to *Staphylococci* than gentamicin for a gentamicin-resistant strain in a developing biofilm [10].The release of anti-cancer therapeutic molecules from nanoporous silica-based SPIONS has been shown to be pH-dependent [11].

The vast majority of bacteria are found in biofilms [12]. Biofilms are microbial consortia embedded in a self-produced matrix of extracellular polymeric substances (EPS) composed mainly of polysaccharides, proteins and extracellular DNA [13]. Studies that examine the bactericidal effects of NPs [14] must therefore take into account the role of the biofilm, including how the size and physicochemical characteristics of the NPs will impact their transport in the biofilms. For example, nanoparticle size has been related to the diffusion through [15][16] and penetration into [17] biofilms. On the other hand, results have been contradictory when examining particle charge effects. For example, little effect of NP charge was observed for the diffusion of functionalized silicon, gold and titanium NPs in a biofilm of *Pseudomonas fluorescens* (*P*. *fluorescens*) [15], whereas in another study on the same biofilm, the observed decrease in the self-diffusion coefficient of carboxylated nano-silver [16] was attributed to its significant negative charge [16].

To our knowledge, no study has been carried out which relates the bactericidal effect of SPIONs to their physicochemical properties. Therefore, the goal of this study was to evaluate the role of particle charge on the penetration and bactericidal efficiency of SPIONs for a biofilm of *Streptococcus mutans*.

## Materials and Methods

### Chemicals

A solution of ferric chloride (FeCl_3_, 45%), ferrous chloride tetrahydrate (FeCl_2_.4H_2_O, >99%), sodium hydroxide, and diethyleneglycol were purchased from Fluka (Belgium). Dimethylformamide (DMF), acetone and diethyl ether were purchased from Sigma-Aldrich (Belgium). 3-(triethoxysilyl)propyl succinic anhydride (TEPSA) and N-[3-(trimethoxysilyl)propyl] ethylenediamine (TPED) were purchased from ABCR (Germany). Ultrafiltration membranes with a molecular weight cutoff (MWCO) of 30,000 g mol^-1^ were purchased from Millipore (USA). Trypticase yeast extract (TYE) medium was prepared using 17 g/liter of BBL trypticase peptone, which contains a pancreatic digest of casein (B11921; BD), 3 g/L of yeast extract (VWR), 5 g/L NaCl (Fisher Scientific) and 2.5 g/L Na_2_HPO_4_ (Sigma-Aldrich). A liquid or solid culture medium (solid medium also contained 15 g/L agarose and 25 mL/L of 40% glucose; Fluka) was obtained. Solutions of 40% glucose and saccharose (Difco) were prepared in Milli-Q water and filtered over a 0.45 μm polycarbonate filter. A phosphate buffered saline (PBS) buffer (pH 7.2) was prepared with 8.76 g/L NaCl (Fisher Scientific), 6.05 g/L K_2_HPO_4_ (Sigma), and 1.7 g/L KH_2_PO_4_ (Sigma) in Milli-Q water. With the exception of the glucose solution for the first 24 h of the culture preparation and the saccharose solution for the second 24 h of the culture preparation, all culture media components (yeast extract, NaCl, Na_2_HPO_4_, tryptone and agar) was dissolved in Milli-Q water and autoclaved prior to use. After autoclaving, the culture media was cooled to room temperature and mixed with the glucose. The medium was poured into petri dishes, solidified and then kept at 4°C until use. Hydrogen peroxide and polyethylene glycol were purchased from Fisher Scientific. Tetrahydrofuran (THF) was purchased from Sigma. The BacLight kit used to quantify cellular viability (Propidium iodide and Syto 9) was purchased from Invitrogen.

### Preparation of nanoparticles

**Fig 1:**
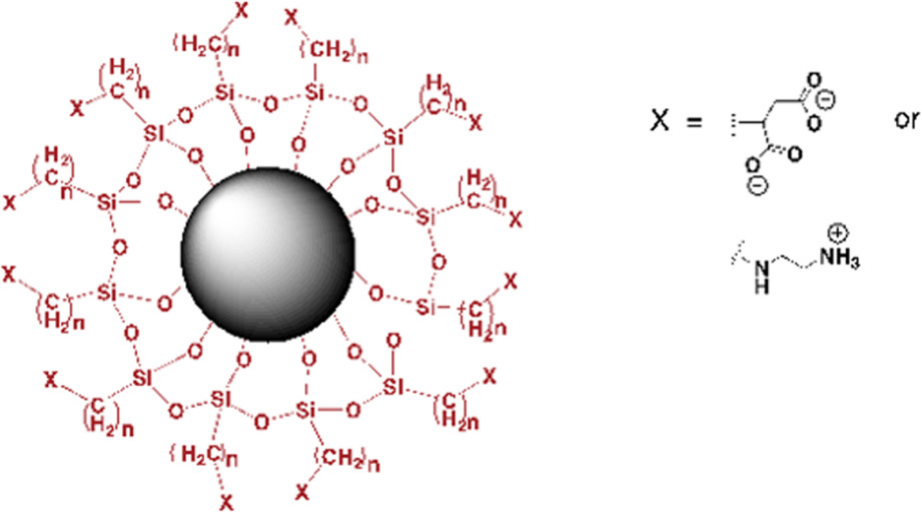
Structure of the charged SPIONs. The amine and carboxyl functional groups are attached to the silica shell on the surface of the SPIONs.

(Fig 1). Bare SPIONs (Fe_3_O_4_): Five mL of an aqueous solution of FeCl_2_.4H_2_O (0.045 M) and FeCl_3_.6H_2_O (0.0375 M) were added to 250 mL of diethyleneglycol. After heating the mixture at 170°C, it was maintained at this temperature for 15 min prior to the addition of base (i.e. solid NaOH to a final concentration of 0.375 M). The temperature was maintained at 170°C for an additional hour before cooling to 60°C. The SPIONs were collected with a neodymium magnet and washed with 100 mL of a 1 M HNO_3_ solution [18].

*Negatively charged (carboxylate functionalized) SPIONs* [19]: 14.2 mL of 3-(triethoxysilyl)propylsuccinic anhydride (TEPSA, 50 mmol) was slowly added to a nanoparticle suspension (100 mM of iron in 100 mL of DMF). 8.6 mL of water, followed by 5 mL of 1 M tetramethylammonium hydroxide were added at room temperature under continuous stirring. This solution was then heated to 100°C for 24 h. The SPIONs were precipitated by addiing a 50/50 acetone/ether mixture prior to their collection using a neodymium magnet. The particles were washed several times with acetone and re-dispersed in water. Excess silanes and other residual chemicals were removed by membrane filtration (30,000 MWCO).

*Positively charged (amino functionalized) SPIONs*: N-[3-(trimethoxysilyl)propyl] ethylenediamine (TPED) was grafted to the SPIONs by adding 25 mmol of TPED (5.4 mL) to a suspension of NP (100 mL, [Fe] = 25 mM) at 50°C. After stirring for 2 h at reflux, the mixture was cooled to room temperature and the suspension was purified by membrane filtration (30,000 MWCO) and centrifugation at 16,500 xg for 45 minutes [18].

### Fourier Transform Infrared (FTIR) Spectroscopy

FTIR spectra were acquired using a Perkin Elmer Spectrum 65 FTIR with an attenuated total absorbance probe in the range of 600–4000 cm^-1^ and a 4 cm^-1^ resolution. Thirty-two scans were combined in order to improve the signal to noise.

### Dynamic Light Scattering

The particle size distribution of the SPIONs was measured in aqueous suspensions using dynamic light scattering (DLS) (Malvern Zetasizer Nano ZS). Samples were sonicated prior to analysis. Scattered light was measured at different angles in the range of 60°–120° at a temperature of 25°C. Intensity distributions were converted into volume and number distributions in order to obtain mean sizes. Means and standard deviations were obtained from three replicate measurements.

### Electrophoretic mobility/zeta potential measurements

A Zetasizer Nano ZSP (Malvern) was used to infer the nature of the charge on the bare, positively charged and negatively charged SPIONs, before and after incubation with the bacterial biofilm.

### Scanning electron Microscopy

A JEOL JSM-7600TFE scanning electron microscope (SEM) was used to obtain photomicrographs at 35,000x. Samples were deposited onto an aluminum substrate. Images were obtained using accelerating voltages of 2 and 5 kV.

### Transmission electron microscopy

A transmission electron microscope (TEM) JOEL JEM 2100F was used to image the SPIONs. Gatan Digital Micrograph software was used for the analysis of the TEM images. Average sizes of the SPIONs were calculated on the basis of fifty NPs for each sample.

### Time-of-Flight Secondary Ion Mass Spectrometry (TOF-SIMS)

Positive and negative ion spectra were obtained using a TOF-SIMS (ION-TOF IV) with a 15 kV Bi^+^ primary ion source, so as to acquire masses up to 100, while maintaining the primary ion dose at less than 10^12^ ions cm^-2^ in order to ensure static conditions. Positive ion spectra were calibrated to the H^+^, C^+^, CH^+^, CH_2_^+^, CH_3_^+^, C_2_H_5_^+^ and C_3_H_5_^+^ peaks and negative ion spectra were calibrated to the C^-^, C_2_^-^, CH^-^, C_2_H^-^, C_3_^-^, C_3_H^-^ peaks before data analysis. Sample spectra were taken over an area of 50 μm ×50 μm, with an emission current of 1.0 μA in bunch mode, rastered in random mode and presented over 128x128 pixels.

### Preparation of the biofilms

*S*. *mutans* (NCTC 10449) was inoculated on solid TYE medium and then incubated for 24 h in the dark at 37°C. A small number of bacterial cells were transferred into 10 mL of the liquid TYE medium containing 0.2% glucose, where they were again incubated for 24 h (37°C, dark). One mL of the bacterial suspension was then diluted 10-fold into liquid TYE medium containing 0.5% saccharose. Five hundred microliters of the mixture was added to each well of an 8-well slide (Nunc), and the culture was incubated for another 24 h in the dark at 37°C in order to produce replicate biofilms (i.e. in each well).

### Bacterial cell counting

The optical density of the bacterial suspensions was measured with a HACH DR 2800 spectrophotometer. Bacterial cell numbers resulting from 10 mL of the liquid TYE medium (containing 0.2% glucose) were determined following 24 h of incubation in the dark at 37°C. Several aliquots of the suspensions at different dilutions were inoculated onto solid TYE medium and incubated for 24 h (dark, 37°C) in order to determine the initial bacterial density.

### Incubation of the SPIONs with the biofilm

The biofilms were gently washed twice with PBS buffer and then incubated with 1 μg mL^-1^, 2 μg mL^-1^ or 3 μg mL^-1^ of the bare, positively or negatively charged SPIONs in the dark for 3 h at 37°C. SPION concentrations were measured by atomic absorption spectrometry (Thermo Scientific).

### Measurements of cell viability with BacLight

Bacteria were exposed to the SPIONs in the 8 well microscope slide. After 4 hours, biofilms were gently washed (2x) with the PBS buffer (pH 7.2) and then incubated with 200 μL of a BacLight kit solution (0.1% Propidium iodide and 0.1% Syto 9 in 0.85% NaCl). Following 15 minutes of incubation at room temperature in the dark, excess BacLight solution was removed and the samples were washed with PBS.

Bacterial viability was determined by counting (minimum of 5 images in each of 3 wells of the microscope slide) stained cells on a confocal laser scanning microscope (Leica TCS SP5) using either excitation by an argon ion laser at 488 nm (emission at 514 nm) or by a diode pumped solid state laser excitation at 561 nm (emission at 580 nm). The measurements were performed *in triplicate*. Average values of cell viability were calculated using the QtiPlot software.

### Analysis of colony-forming units

For the analysis of colony-forming units (CFU), triplicate samples of the *S*. *mutans* were incubated with the bare, positively charged and negatively charged SPIONs for 3h. Biofilms were removed from the bottom of petri dishes by scraping them quantitatively with pipette tips. Cell suspensions were transferred into an Eppendorf tube and dispersed by sonication for 30 s. Cell viability was expressed as CFU per mL and compared with cells that had not been exposed to the SPIONs (controls).

## Results and Discussion

### Physicochemical characterization of the SPIONs

**Fig 2:**
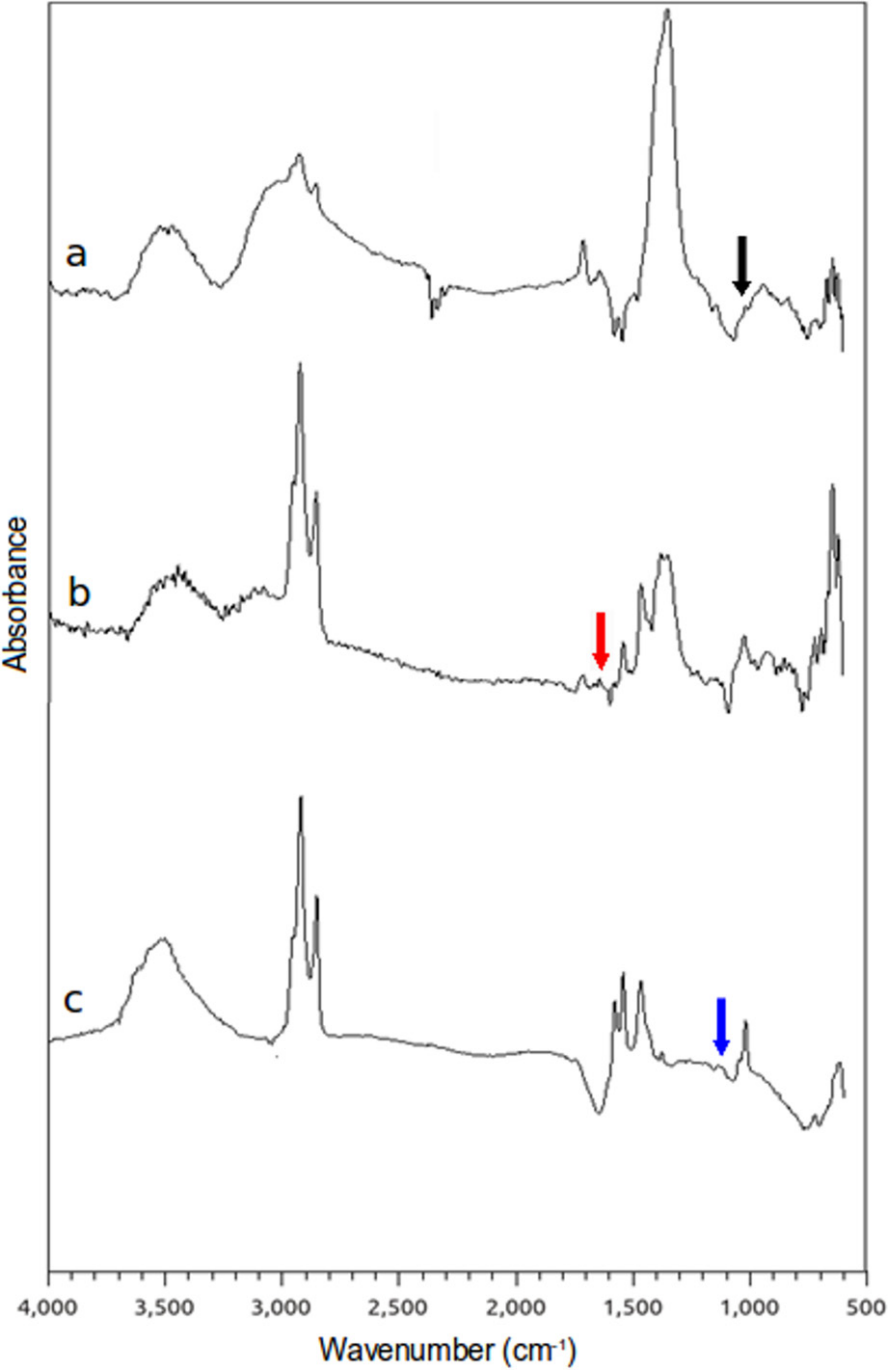
**FTIR spectra of a) bare, b) positively and c) negatively charged SPIONs.** In Fig 2a, the peaks at 1000 cm^-1^, 1100 cm^-1^ and 1660 cm^-1^ are indicated by black, blue and red arrows, respectively.

Fig 2 shows the FTIR spectra of the three SPIONs. Peaks at 635 cm^-1^, 1000 cm^-1^ and the strong band at 1330 cm^-1^ are attributed to Fe-O stretching, C-O stretching and CH_2_-OH stretching, respectively (Fig 2A, 2B and 2C). The broad band at 3400–3500 cm^-1^ corresponds to the presence of O-H stretching, due to hydration water. The Si-O stretching bond, which is due to the silica shell on the surface of positively and negatively charged SPIONs, appears at around 1100 cm^-1^ [20] (Fig 2B and 2C). The peak that appears near 1660 cm^-1^ in the FTIR spectra of positively charged SPIONs corresponds to the amine bending mode [21] (Fig 2B), whereas the peaks at 1555 cm^-1^ and 1420 cm^-1^ can be attributed to asymmetric and symmetric stretching of COO^-^ [22] (Fig 2C).

**Fig 3:**
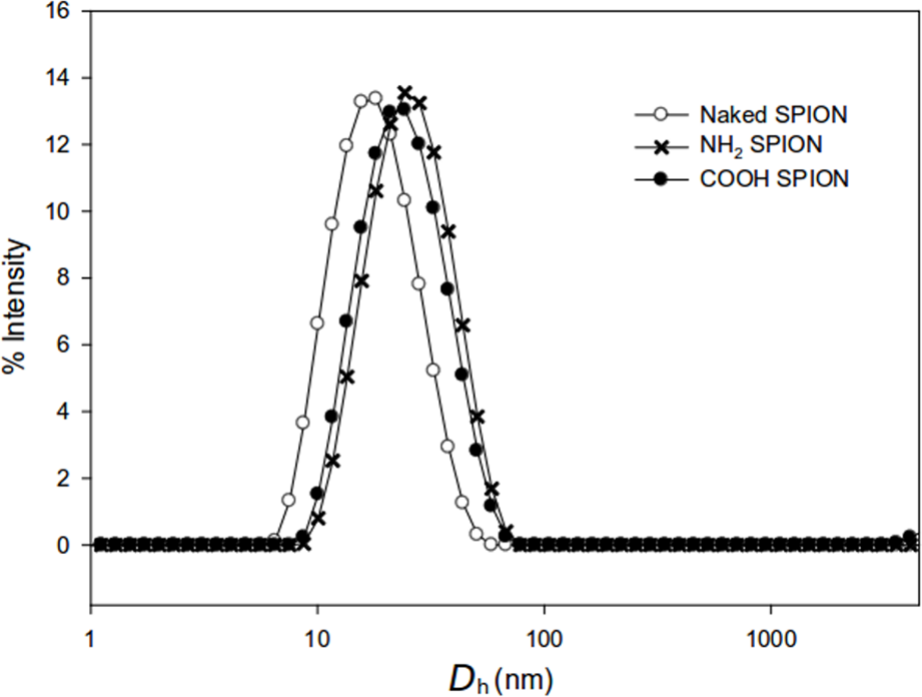
**Particle size distributions of the a) bare, b) positively and c) negatively charged SPIONs.** The charged SPIONs, due to their silica shell, were slightly larger than the bare ones.

Hydrodynamic diameters of the SPIONs measured by DLS were fairly similar: 14.6 ± 1.4 nm for the bare SPIONS, 20.4±1.3 nm for the positively charged SPIONS and 21.2 ± 1.6 nm for the negatively charged SPIONs (Fig 3).

Electrophoretic mobilities of the SPIONs in the PBS buffer (pH 7.2) were measured before and after incubation with the bacterial biofilms (Table 1). The bare SPIONs exhibited a small positive ζ potential (5.9 mV). As expected, the amino functionalized SPIONs had a large, positive ζ potential (22.2 mV) while the carboxylated SPIONs had a substantial negative ζ potential (-20.1 mV). Interestingly, incubation with the biofilms resulted in leveling of the mobility values, with all SPIONs showing negative mobilities following incubation with the biofilms. This behavior is consistent with the surface adsorption of the bacterial exopolymers on the surface of the SPIONs. Although the biofilm is globally negative charged, van der Waals and hydrophobic forces can be sufficiently large to explain the adsorption of the negatively charged biopolymers to the negatively charged polymer surface. The observed zeta potentials between -15 and -17.2 mV are likely sufficient to stabilize the colloidal suspensions of SPIONs.

**Table 1:** Zeta potential values of the SPIONs determined before and after incubation with the biofilm.

Samples	ζ potential (mV) before incubation with biofilm	ζ potential (mV) following incubation with biofilm
Bare SPIONs	5.9 ± 0.5	-17.2 ± 0.7
Amino functionalized SPIONs	22.2 ± 0.3	-15 ± 0.5
Carboxyl functionalized SPIONs	-20.1 ± 0.4	-16.9 ± 0.6

**Fig 4:**
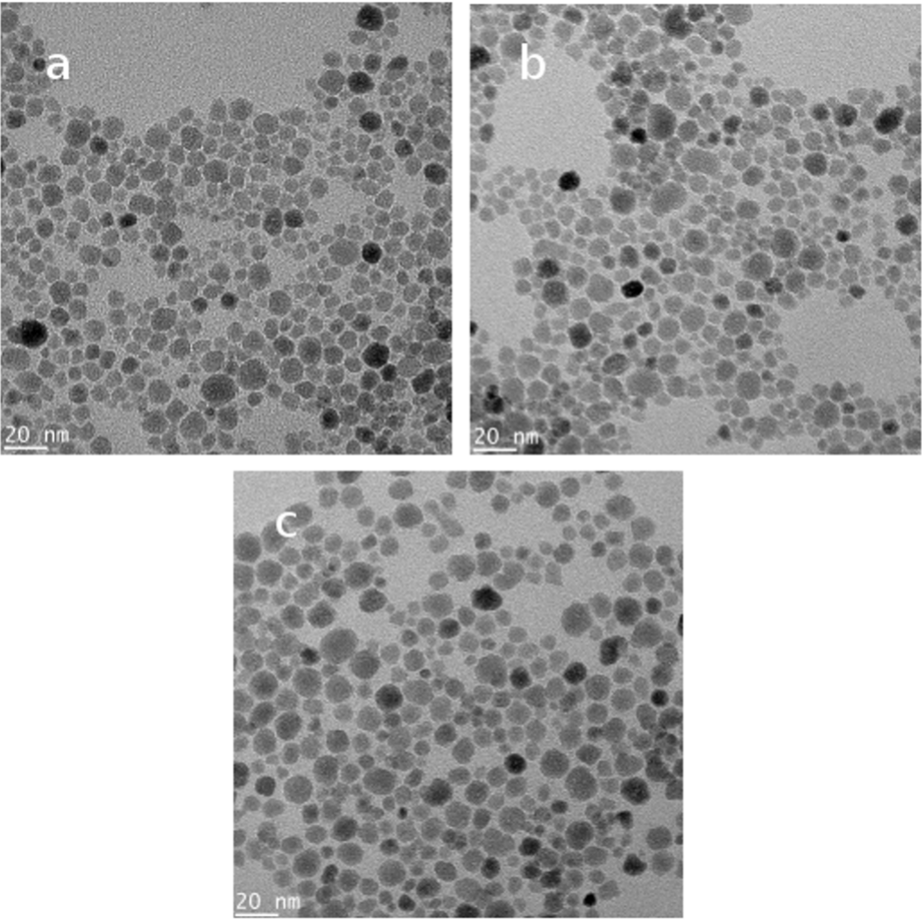
TEM images of (a) bare, (b) positively charged and (c) negatively charged SPIONs.

**Fig 5:**
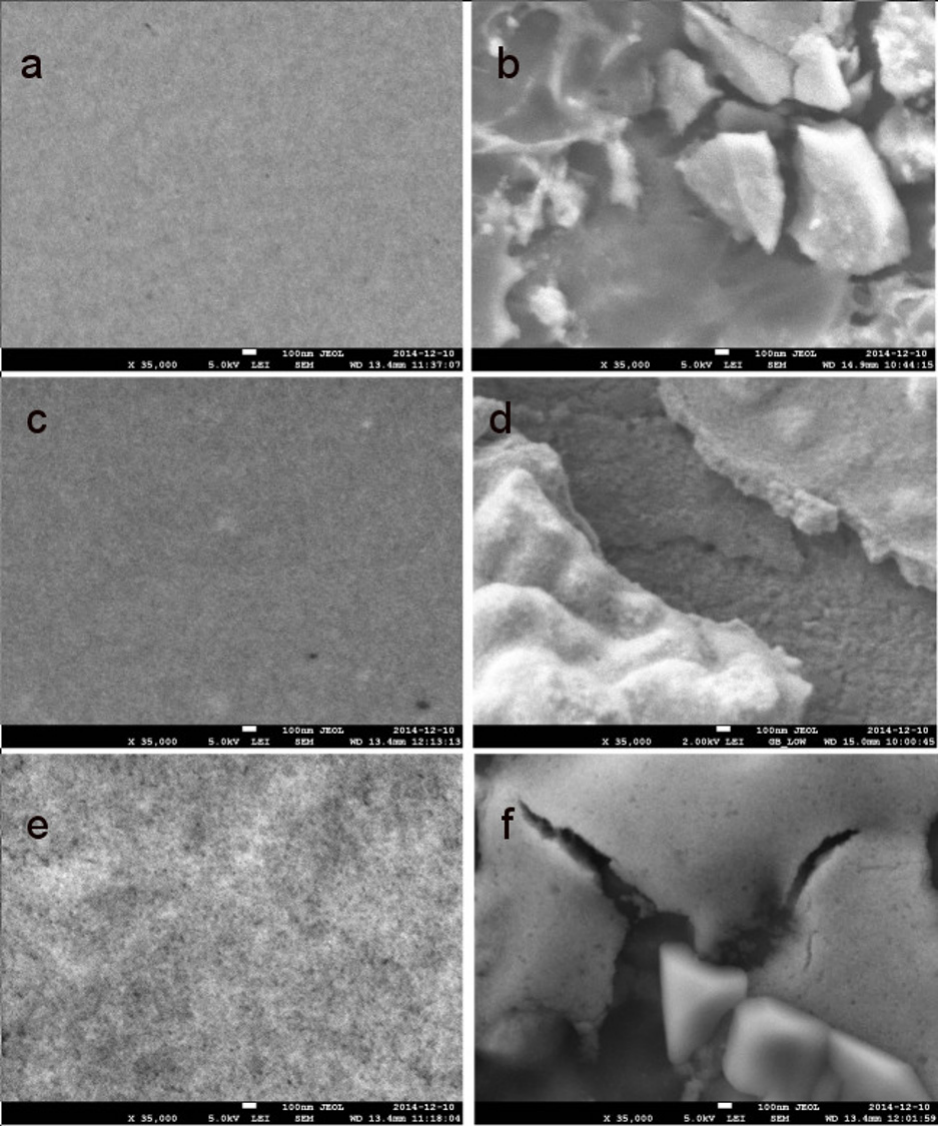
**SEM images of (a,d) bare, (b,e) positively charged and (c,f) negatively charged SPIONs before and after incubation with the biofilms.** Following incubation with the bacterial biofilms, the SPIONs appeared to be coated with soft matter.

Fig 4 shows the morphology and the sizes of SPIONs obtained using TEM. Size distributions were calculated on the basis of fifty randomly selected NPs. SPIONs were fairly spherical with average diameters of the bare, positively charged and negatively charged SPIONs of 10.1 ± 0.6 nm, 11.4 ± 0.4 nm and 12.1 ± 0.5 nm, respectively. These physical diameters are in good agreement with the hydrodynamic diameters determined by light scattering (theoretically, we expect hydrodynamic diameters to be slightly larger than the physical diameters, [23]). SEM images were also acquired for the bare, positively and negatively charged SPIONs, before (Fig 5A, 5C and 5E) and after (Fig 5B, 5D and 5F) incubation with the biofilms. Diameters were in line with both TEM and DLS: 15.3 ± 1.2 nm (bare, Fig 5A), 18.8 ± 1.5 nm (positively charged, Fig 5C) and 19.3 ± 1.4 nm (negatively charged, Fig 5E).

Fig 5 also shows the presence of large quantities of soft matter, likely corresponding to the adhesion of the bacterial EPS. An increase in particle size following incubation with the biofilms is observed in Fig 5B, 5D and 5F, likely due to the adhesion of biomolecules to the nanoparticles.

**Fig 6:**
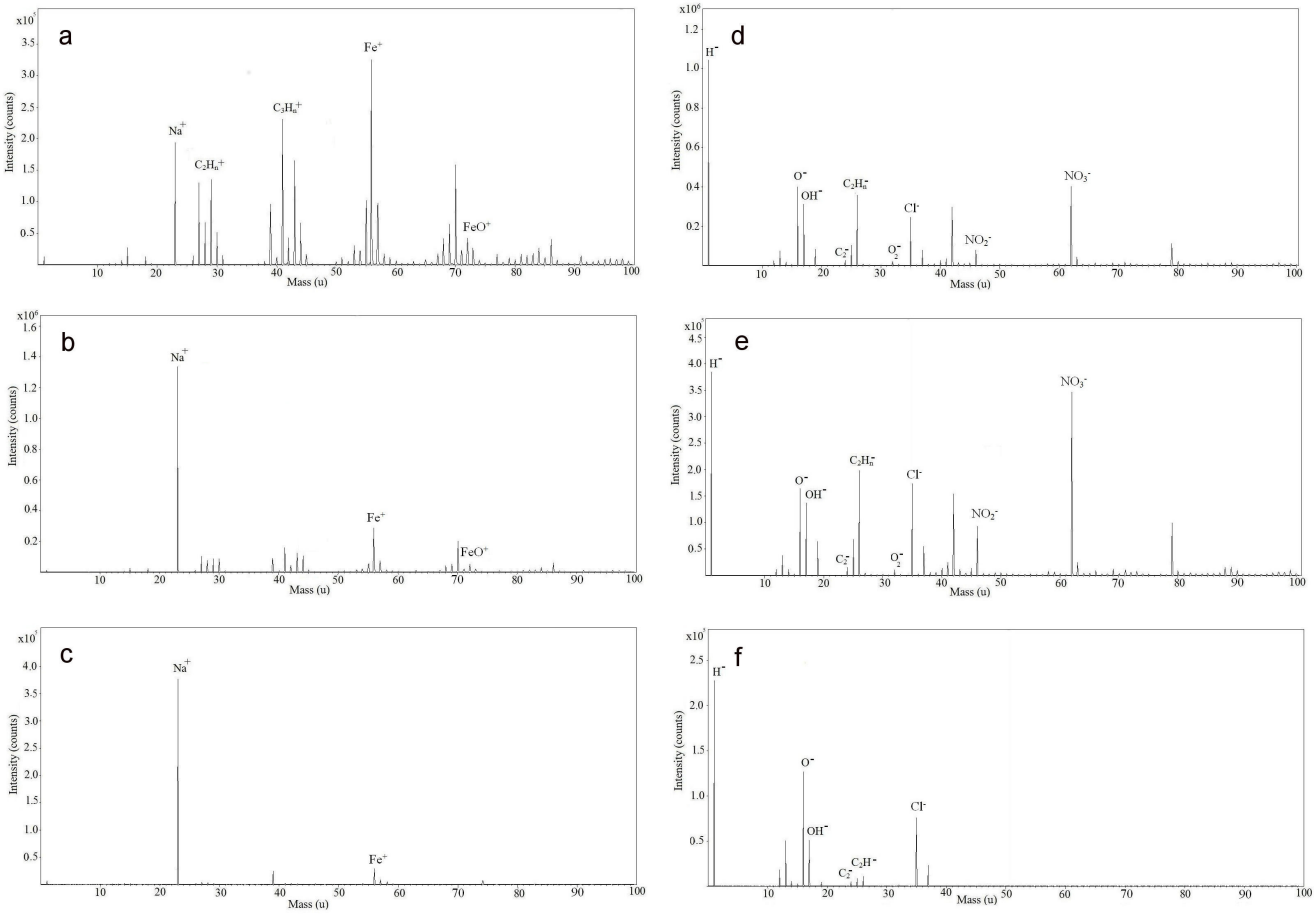
**Positive TOF-SIMS spectra of (a) bare, (b) positively charged and (c) negatively charged SPIONs; negative TOF-SIMS spectra of (d) bare, (e) positively charged and (f) negatively charged SPIONs after incubation with the biofilm.** The intensity of each peak was different for each sample. The intensity peak ratios changed from one sample to the others.

TOF-SIMS spectra of the bare, positively and negatively charged SPIONs before incubation with the biofilms are provided in S1 Fig. Following their incubation with the biofilms, mass spectra of the bare, positively and negatively charged SPIONs were reacquired (Fig 6). In positive mode, the primary peaks that were observed corresponded to: Na^+^ (m/e 23), Si^+^ (m/e 28), SiO^+^ (m/e 44), Fe^+^ (m/e 56), FeO^+^ (m/e 72), FeOH^+^ (m/e 73), as well as some weak hydrocarbon peaks. In negative mode, peaks corresponding to O^-^ (m/e 16), OH^-^ (m/e 17), O_2_^-^ (m/e 32), Cl^-^ (m/e 35 and 37), NO_2_^-^ (m/e 46) and NO_3_^-^ (m/e 62) were observed.

The variations in the intensities of the NH_2_^+^ and the COOH^-^ peaks provided some information on the amine and carboxyl functional groups on the surfaces of the charged SPIONs. As expected, the intensity of the NH_2_^+^ peak for the positively charged SPIONs was higher than that of the bare and negatively charged SPIONs. Similar observations were made based upon the carboxylate peaks where the negatively charged particles clearly had a greater COOH^-^ intensity than either the bare (14x) or the positively charged (1.5x) SPIONs. Nonetheless, the measured intensities on the functionalized SPIONs were greatly attenuated in the presence of the biofilm (40% attenuation for the bare SPIONs; 60% attenuation for the positively charged SPIONs). The decrease in the intensities of COOH^-^ peak was also observed in the spectra of the negatively charged SPIONs after incubation with the biofilm (20%). On the other hand, the intensities of the NO_2_^-^ and NO_3_^-^ peaks in the spectra of the positively charged SPIONs increased after incubation with the biofilm (1.3x for the NO_2_^-^ peak and 1.8x for the NO_3_^-^). The increased intensity in the presence of the biofilm was attributed to interactions of the SPIONs with the biofilm components.

### Quantification of the bacteria

Based upon optical density measurements, there were initially 10^7^ bacteria per mL of liquid TYE medium following incubation for 24 h in the dark at 37°C. This liquid broth was used to seed the bacterial biofilms. Two controls were evaluated for the confocal microscopy measurements: (i) biofilms without the SPIONs and without the stains of the BacLight kit and (ii) biofilms without the SPIONs but with the stains of the BacLight kit. No autofluorescence was observed for the bare, positively or negatively charged SPIONs (data not shown). The average thickness of the biofilms was 15.0 ± 0.5 microns.

**Fig 7:**
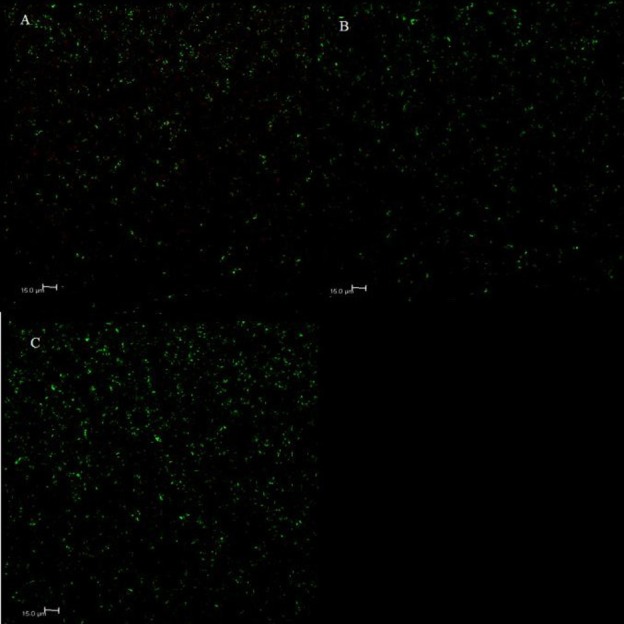
**Representative images of the bactericidal effect of (A) bare, (B) positively charged and (C) negatively charged SPIONs on the *S*. *mutans* biofilm for a SPION concentration of 2 μg mL**^**-1**^. Images for exposures to 1 μg mL^-1^ and 3 μg mL^-1^ can be found in S2 and S3 Figs. For each image, green coloration indicates live cells while red coloration indicates dead cells.

Bare, positively charged or negatively charged SPIONs were added to triplicate biofilms of *S*. *mutans*. The proportion of dead (red) cells to total cells was significantly higher in the biofilms exposed to the SPIONs than in their absence. Nonetheless, the bactericidal effect of SPIONs was not the same in different spots on the biofilm. Therefore, biofilm heterogeneity was taken into account by quantifying large numbers of cells over several independently prepared surfaces (Fig 7). The proportion of dead to total cells following 3 hours of incubation of the SPIONs with the biofilms is given in Table 2. For 1 μg mL^-1^, larger proportions of dead cells were observed for the bare SPIONs or positively charged SPIONs in comparison to the negatively charged SPIONs at the same concentration. When 2 or 3 μg mL^-1^ of SPIONs were incubated with the biofilm, toxicity was the highest for the positively charged SPIONs followed by the bare and then the negatively charged SPIONs. In all cases, toxicity increased with concentration and significantly more bacteria were killed in the presence of the positively charged SPIONs as compared to the negatively charged SPIONs. This result can be attributed to an increased electrostatic repulsion between the carboxylated surface of the SPIONs and the globally negatively charged polymer matrix of the biofilm. While the observations in the biofilms were the result of the averaging of numerous heterogeneous zones of differing response, average results determined by scraping large zones of biofilm were consistent with the confocal images. For example, when CFU/mL values were determined for the bacteria in solution- significantly higher cell numbers were observed for control cells as compared to those incubated with the SPIONs and toxicity appeared to decrease in the order: positively charged SPIONs>bare SPIONs>negatively charged SPIONs (Table 3).

**Table 2:** The proportion of dead cells to total cells in the *S*. *mutans* biofilms following 3h of incubation with the bare, positively charged and negatively charged SPIONs. Data were obtained after correction for the number of dead cells in the control images (stained bacteria without SPIONs), which was 10% ± 5%. N = 15.

Concentrations of SPIONs (μg mL^-1^)	Bare SPIONs	Positively charged SPIONs	Negatively charged SPIONs
1	9% ± 2%	10% ± 2%	3% ± 2%
2	14% ± 2%	26% ± 3%	11% ± 3%
3	25% ± 2%	32% ± 2%	19% ± 3%

**Table 3:** CFU values (/mL) for *S*. *mutans* isolated from biofilms that were either exposed to bare, positively charged or negatively charged SPIONs or were incubated in the absence of SPIONs.

Concentrations of SPIONs (μg mL^-1^)	Bare SPIONs	Positively charged SPIONs	Negatively charged SPIONs	Control
1	6.8 x10^5^	6.6 x10^5^	9.2 x10^5^	1.6 x10^6^
2	5.7 x10^5^	5.5 x10^5^	7.2 x10^5^	1.7 x10^6^
3	5.3 x10^5^	4.5 x10^5^	6.6 x10^5^	1.7 x10^6^

The literature on the bactericidal effect of SPIONs on biofilms is relatively sparse. Our results indicate that toxicity decreases in the order: positively charged>>negatively charged SPIONs and is dose dependant. Given that the sizes of the variable charged particles are similar, especially with respect to the average pore size of the biofilm that is thought to be around 50 nm [24], differences in particle toxicity can be attributed either to the particle functionalization or particle charge. Given that the charges on most biofilms [25] and most biological membranes are negative [26], we anticipated that the positively charged particles would more easily penetrate into the biofilms, leading to increased toxicity. Indeed, an electrostatic repulsion of the negatively charged particles, leading to less particle penetration and less diffusion, is consistent with the observations of reduced toxicity for the carboxylated SPIONs. Nonetheless, the electrophoretic mobilities of the SPIONs following a short (3 hour) incubation with the biofilms showed that they all had very similar zeta potentials. We speculate that the significant differences in toxicity may have been due to the kinetics and/or magnitude of the surface modification, i.e. either the adsorption of the biofilm components was slower than the penetration into the biofilm or the concentration of biopolymers was less important in the biofilms than in the culture medium. As expected, the CFU/mL values of non-incubated (control) *S*. *mutans* were more than those of incubated bacteria with the NPs. The CFU/mL values of *S*. *mutans* were significantly influenced by concentrations of SPIONs and were dose-dependent. Our results indicate that the CFU/mL values of *S*. *mutans* incubated with the SPIONs decreases in the order: negatively charged>>bare SPIONs>>positively charged SPIONs. This means that more bacteria are alive when they are incubated with less toxic SPIONs. The analysis of the CFU/mL values confirms our data on the bactericidal effect of SPIONs on *S*. *mutans* biofilm and shows that the SPIONs have the same toxicity on bacteria in solution in comparison with that on the biofilm.

Biofilm biopolymers adhere favorably to the surface of NPs due to their high surface to volume ratio (S/V) and high surface potential [27]. The proteins are known to form protein corona on the surface of NPs [28], leading to a modification of their physicochemical nature, perhaps substantially different for the carboxylated as opposed to the amino functionalized SPIONs. TOF-SIMS indicated differences among three SPIONs incubated with the biofilms (Fig 6), showing that although overall surface potential was likely similar, surface functionalization was different for the three SPIONs, especially for the carboxylated SPIONs. More investigation is required to determine which EPS proteins attach to the surface of SPIONs and at what rate following their incubation with the biofilm. Examination of the toxicity mechanisms (e.g. Reactive oxygen species (ROS) generation) may also be useful to distinguish the biological effects of these nearly identical core particles.

## Conclusions

This study verified the antibacterial effect of three variably charged SPIONs. Sizes and surface properties of the SPIONs were characterized before and after incubation with a bacterial biofilm. Although the clear differences in particle surface properties were attenuated following incubation with the biofilm, based upon the toxicity results, it is likely that subtle differences remained or that surface modifications were not instantaneous in the biofilm and the observed differences in toxicity could be attributed to the initial differences in particle charge. Nonetheless, our data suggest that the surface properties, as opposed to the core properties of the SPIONs, are critical for determining NP toxicity. In the future, more investigations would certainly be helpful in order to get a more in depth understanding of the biochemical mechanism(s) of the different SPIONs.

## Supporting Information

S1 Fig Positive TOF-SIMS spectra of (a) bare, (b) positively charged and (c) negatively charged SPIONs; negative TOF-SIMS spectra of (d) bare, (e) positively charged and (f) negatively charged SPIONs before incubation with the biofilm.
(TIF)

S2 Fig **Representative images of the bactericidal effect of (A) bare, (B) positively charged and (C) negatively charged SPIONs on *S*. *mutans* biofilm for a SPION concentration of 1 μg mL^-1^.**
(TIF)

S3 Fig **Representative images of the bactericidal effect of (A) bare, (B) positively charged and (C) negatively charged SPIONs on *S*. *mutans* biofilm for a SPION concentration of 3 μg mL^-1^.**
(TIF)
