# Supplementary figures and images for: Relating the Surface Properties of Superparamagnetic Iron Oxide Nanoparticles (SPIONs) to Their Bactericidal Effect towards a Biofilm of Streptococcus mutans
Source: PLoS One. 2016 Apr 26;11(4):e0154445. doi: 10.1371/journal.pone.0154445 (PMC4845983; doi:10.1371/journal.pone.0154445)

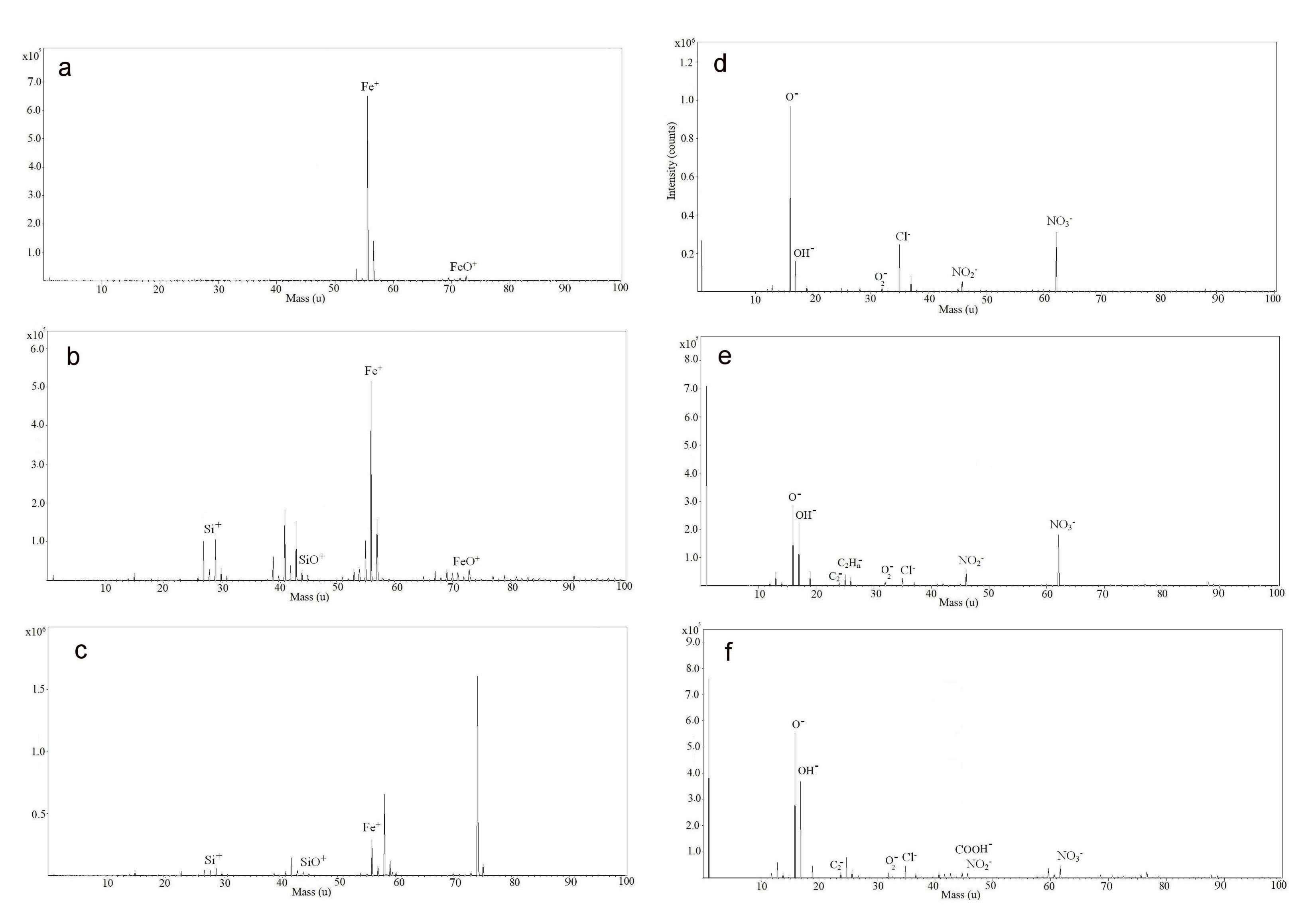

Supplement: S1 Fig — Positive TOF-SIMS spectra of (a) bare, (b) positively charged and (c) negatively charged SPIONs; negative TOF-SIMS spectra of (d) bare, (e) positively charged and (f) negatively charged SPIONs before incubation with the biofilm. (TIF) [file pone.0154445.s001.tif]

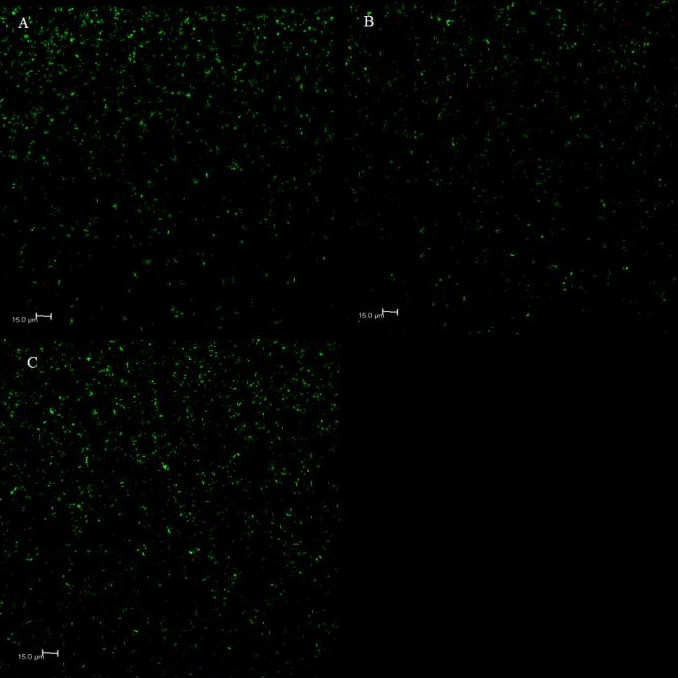

Supplement: S2 Fig — Representative images of the bactericidal effect of (A) bare, (B) positively charged and (C) negatively charged SPIONs on S. mutans biofilm for a SPION concentration of 1 μg mL-1. (TIF) [file pone.0154445.s002.tif]

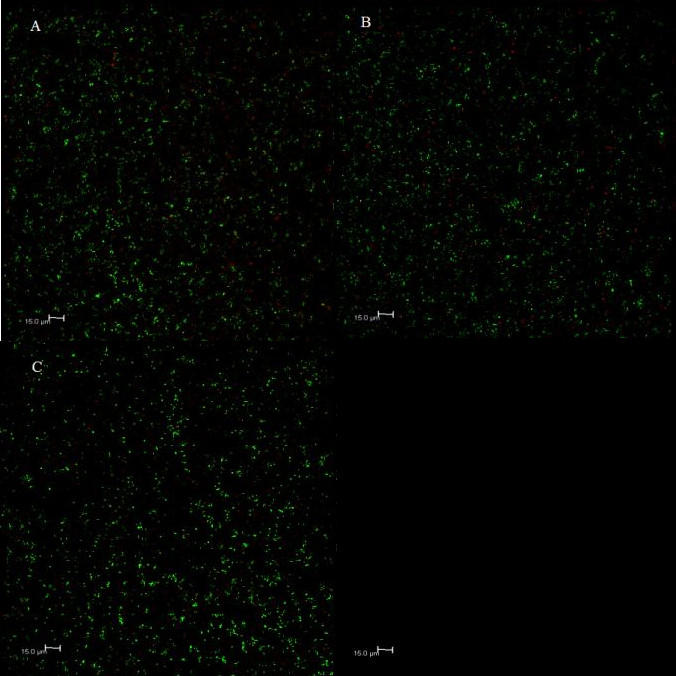

Supplement: S3 Fig — Representative images of the bactericidal effect of (A) bare, (B) positively charged and (C) negatively charged SPIONs on S. mutans biofilm for a SPION concentration of 3 μg mL-1. (TIF) [file pone.0154445.s003.tif]
